# Reusability challenges of livestock production data to improve animal health

**DOI:** 10.1038/s41597-025-04785-4

**Published:** 2025-03-19

**Authors:** Camille Delavenne, Gerdien van Schaik, Jenny Frössling, Angus Cameron, Céline Faverjon

**Affiliations:** 1 EpiMundi, Lyon, France; 2 Department of Population Health Sciences, section Farm Animal Health, Faculty of Veterinary Medicine, Utrecht University, Utrecht, the Netherlands; 3 Royal GD, Deventer, the Netherlands; 4 Department of Epidemiology, Surveillance and Risk Assessment, Swedish Veterinary Agency (SVA), SE-751 89 Uppsala, Sweden; 5 Department of Applied Animal Science and Welfare, Swedish University of Agricultural Sciences (SLU), Box 234, SE-532 23 Skara, Sweden

**Keywords:** Agriculture, Research data

## Abstract

In veterinary epidemiology, using data routinely generated by stakeholders of the livestock production chains offers an opportunity for researchers to access a large amount of information that could be used to improve animal health. However, (re)using these non-scholarly data doesn’t come without challenges. This study assesses the reusability for research purposes of 30 European datasets generated by the livestock sector to meet legislative or operational needs. Information about each dataset was collected through a questionnaire survey filled by the data owner or the data user (researchers). Datasets were described, and their compliance with the FAIR principles, a data-sharing standard, and the principle of accountability defined in the General Data Protection Regulation were assessed. The study highlighted major gaps in terms of compliance with data regulations and implementation of good data management practices, specifically considering the rare use of metadata and standard vocabularies. Filling these gaps is essential to reap the full benefits offered by the rapidly growing volume of heterogeneous data available in livestock production systems.

## Introduction

Policymakers, funders and other stakeholders are currently investing considerable efforts to promote good data stewardship to facilitate data access and reuse and thus leverage investments in research, improve research reproducibility, and advance innovation^1–5^. In that context, data stewardship means not only proper data collection and annotation but also the ‘long-term care’ of the data to preserve them for future uses^6^. As part of these efforts, the FAIR guiding principles^6^ have been developed to support stakeholders in the improvement of their data stewardship practices and are becoming a cornerstone of research policy and requirements for research data management plans (e.g., European research programs^7,8^). They are built on four foundational principles (i.e., Findability, Accessibility, Interoperability and Reusability) and primarily target scholarly data, meaning datasets used for research with the intention of producing a scholarly publication. However, the dream of scholarly data being easily reusable has yet to come true and significant improvements are still needed in many disciplines^2,9–12^.

The veterinary epidemiology domain is very broad but is mostly concerned with the distribution and determinants of animal health, welfare and production. Those determinants are used as a basis to better control and prevent health problems to improve animal production sustainability and food security. The raw data used in veterinary epidemiology studies are not always collected directly by researchers for the purpose of research but also by public or private livestock value chain stakeholders to meet regulatory or operational needs (e.g., diseases notification system, management of production performances, health records, animal movements). Many of these data sets are or are becoming “big” data^13^ and offer an important opportunity for researchers to access a large amount of information that could be used to improve animal health, welfare, production efficiency and the sustainability of livestock production systems. Moreover, they can provide the information required to better manage complex animal health issues such as unspecific contagious animal diseases (e.g. leading to mortality in farmed fish, respiratory or digestive syndromes in terrestrial production species), which are still poorly understood even though they have been identified as keys to improve antimicrobial stewardship and strengthen animal production and welfare^14^. As these data are often data streams, they also have the potential to provide near real-time information to support evidence-based decision-making of various stakeholders (i.e., veterinarians, producers, and governmental agencies).

If reusing ‘non-research’ data in a research framework is very tempting, it does not come without challenges. Data reuse is only possible when good data stewardship practices make the data findable, accessible, interoperable or even reusable, but it requires skills and resources. The challenge of implementing good data stewardship has been highlighted as a potential reason explaining the limited adoption of the FAIR principles in veterinary epidemiology^15^. However, for the private sector, making data accessible, reusable or even findable by others is usually considered a threat rather than an opportunity^16–18^. Because the private sector is subject to market pressures, it is usually best not to facilitate access to data, which may inadvertently provide an advantage to a competing organisation or be misused^5^. Moreover, these data usually contain ‘personal data’, meaning any information related to an individual who can be directly or indirectly identified^19^ (e.g., identification of the farm sending animals to a slaughterhouse or buying animals from another farm). Personal data are increasingly subject to strict regulations to protect privacy and security for individuals and businesses. For this purpose, the European Union (EU) defined the EU General Data Protection Regulation (GDPR), which applies to any dataset containing personal data. This regulation has been identified as a barrier to data sharing^11,15,20,21^ as it requires that personal data be only collected for a specific purpose, therefore limiting secondary use and potential data integration at an individual level^14^.

Data coming from the private sector are, by nature, very different from scholarly data as they are not usually intended to be shared or reused. However, when used for research, they are, by definition, being shared with and reused by a third party (i.e., researchers). Sharing data between entities means that the data recipient must be able to understand the nature of the data they are receiving, for example, the data quality, to assess whether they may be used to serve the intended purpose. Understanding data created by others can be challenging as each group of stakeholders tends to use its own vocabulary, which is rarely documented and often based on local practices or needs^22^. The diversity of data management practices and tools also makes the understanding of the actual value of the data from a research perspective problematic^8,23^. Being able to reuse these data in a research framework depends thus on the capacity of ‘non-research’ data to meet some data sharing standards (e.g., FAIR guiding principles) even though they should, in theory, neither be expected nor required to meet these standards as they have a different primary purpose than to be shared or reused.

This study aimed to assess how data produced by stakeholders of the terrestrial and aquatic livestock production chain can be reused in a research framework and to investigate how they meet some essential criteria for data sharing and reuse. The case study was based on European datasets generated by public or private organisations to meet regulatory or operational needs. These datasets were accessed by members of a single research consortium and information about them were collected through a questionnaire survey. The datasets included in the study are first described, and their compliance with two data-sharing standards (i.e., FAIR principles and GDPR) is then assessed. FAIR principles were selected as they are internationally endorsed standards to assess how easily these data can be shared and reused. Our study focused mostly on the principles related to good data management practices and ignored those considered not relevant for non-scholarly datasets. The compliance with GDPR was also investigated as the datasets included in the study were part of the European ecosystem.

## Method

### Study population

The datasets included in this study were those intended to be used in a large Horizon 2020 European-commissioned research project named DECIDE. All the datasets were initially created by stakeholders for non-research purposes, but all had attracted the interest of researchers to use them for research.

This project aims to support the development of data-driven tools for decision support to animal health practitioners (veterinarians, farmers, technicians, and others) in the four main production species (poultry, pig cattle and salmonids) (www.decideproject.eu). Because the project is implemented by a large consortium of 19 partners from the public and private sectors across 11 European countries, it was expected to gather many routinely collected ‘non-research’ animal health datasets across Europe, such as data provided by slaughterhouses, diagnostic laboratories, producers (e.g., production performance, mortality, veterinary treatments, etc.) or governmental agencies (e.g., populations, animal movements, costs). The survey was shared with all DECIDE partners, but targeted emails were sent to researchers who had reported working within the project with data available before the start of the project. The researchers were asked to share the survey with the data owner or, when it was not possible, answer the survey themselves.

### Survey design

An online survey was designed to collect information about the datasets and assess their FAIR and GDPR’s principle’s compliance. The survey, made of 103 questions in English, was designed on the online software Limesurvey© (https://www.limesurvey.org/en/). Before being deployed, it was tested by four researchers in epidemiology, including native and non-native English speakers with different levels of knowledge in data management.

The questions were organised into 11 categories: Respondent profile (8 questions), the dataset’s basic information (10 questions), governance (15 questions), management (14 questions), model (11 questions) *(‘data structure’ in the survey)*, description (11 questions*) (‘data subject’ in the survey)*, metadata (defined as all elements describing the data^6^) (15 questions), sharing datasets (12 questions), sharing further information with survey creator (2 questions), sharing expectation (1 question), and survey consent (4 questions). Three main sources of information were used to design the questions: (i) standardised metadata schemas related to precision agriculture (including aquaculture) data and livestock farming^24^ and data catalogue vocabulary (https://www.w3.org/TR/2024/CR-vocab-dcat-3-20240118/), (ii) FAIR principles and the FAIR implementation profile mini questionnaire^6,25^ and (iii) GDPR regulation^19^. Existing metadata schemas were used as a basis to design the questions aiming at describing the content and structure (or metadata) of the datasets, in particular in the questions related to the categories “model”, “description” and “metadata”. In this study, metadata was defined as all elements describing the data^6^. This definition was also made available to the survey respondents at the beginning of the survey. The other sources of information were used to design the questions assessing whether the datasets met the FAIR and the GDPR data protection principles. These questions were mostly related to the categories “governance”, “management”, “sharing datasets” and “metadata”. The full survey is available in the supplementary information document.

### Data collection

The data collection was divided into two survey rounds: A first round was conducted between February and April 2022, and a second one was conducted between March and July 2023. The two rounds were similar, and the second one mostly aimed at adding information related to newly available datasets. The second round also offered the opportunity for the respondents to validate the answers they provided for the first round. The data management and analysis followed the same process as for the two survey rounds. When a dataset had an answer for each round by two different respondents due to miscommunication (and not because of correction needed), the two answers were aggregated. When conflicts appeared, the most coherent response was kept.

### Data cleaning and quality check

Incomplete answers were removed as well as the answers related to publicly available meteorological datasets. These datasets were excluded because they were generated by a different scientific community (ie., researchers in climate) and did not contain information directly related to animal health. They were thus considered out of scope for this study. The remaining answers were then anonymised in two steps. First, a random identification number (ID) was assigned to each dataset and another to each respondent. Then all columns containing identifying information were either removed (question not included in the results) or reclassified to retain only the information relevant to the analysis. For example, the language into which the dataset is available became ‘English’ or ‘not English’. Similarly, if the name of the data owner was provided, the answer was reclassified as “there is an identified data owner”. The processed results are available at Delavenne *et al*.^26^. They include the link to the question of the survey and the main process used to select and retrieve information for each column. Furthermore, the processed results can be linked to all figures and supplementary material using the assigned IDs.

The quality of the answers provided for open questions was assessed for each respondent based on two criteria: completeness and coherence. Each was assessed using a 2-level scoring system (1 or 0). Completeness was assessed by the presence or absence of an answer to the open questions. Coherence was assessed by the relevance of the answers according to the question scope and the complementary information available either directly within the survey itself (e.g., answers provided to other questions or data/metadata shared by the respondent) or from external resources. External sources included the dataset’s metadata and the respondents themselves when they provided complimentary information via emails or face-to-face meetings. It also included the results of an extensive online search based on the dataset’s name, data owner names, or the dataset’s URL when available. When the answer wasn’t coherent, a low score was given (i.e., equal to 0), and the survey answer^26^ was modified to include the correct information.

A global quality grade was then computed for each question considering all the respondents’ answers to that question. It was defined as the sum of the quality scores (i.e., completeness and coherence) actually obtained by all the respondents for that question divided by the maximum quality scores this question could have obtained if all the respondents’ responses were complete and coherent. A quality grade was considered low when it was less than 75%.

### Assessment of GDPR’s accountability principle’s compliance

Compliance with the GDPR regulation was only assessed for the datasets including personal data, as they are the focus of this regulation^19^. Compliance with GDPR is expected to be reached when seven data protection principles are respected: (i) Lawfulness, fairness, and transparency, (ii) Purpose of limitation, (iii) Data minimisation, (iv) Accuracy, (v) Storage limitation, (vi) Integrity and confidentiality, and (vii) Accountability. The first six principles are related to data management processes and are under the responsibility of the data controller, who is the person accountable for compliance with the first six principles^19^. Therefore, in this study, it was considered that the minimum requirement to comply with the GDPR was to be able to properly identify the persons accountable for data management. Data management processes were not assessed. The evaluation was based on two criteria: (i) identification of the two data governance roles required in the GDPR (i.e., data subject and data controller), and (ii) formal documentation of these roles. These criteria were assessed based on a 2-level scoring system (1 and 0) detailed in Table 1.

**Table 1:** GDPR’s accountability principle’s compliance criteria focused on the concept of ‘accountability’ and the corresponding scoring scheme.

Criteria	Score and associated definition
Identified roles	1 = All the data governance roles are identified.
	0 = At least one of the data governance roles is not identified.
Documented roles	1 = All the data governance roles are documented.
	0 = At least one of the data governance roles is not documented.

### Assessment of the FAIR compliance

FAIR guiding principles provide a continuum of increasing reusability and, therefore, do not specify technical requirements^27^. However, they describe characteristics and aspirations, allowing research outputs to be transparent and extensively reusable to benefit all. However, although machine readability is central to FAIR guidelines, not all data should or could be machine-actionable^4,27^. In some cases, the appropriate technology is unavailable, or the human or financial resources are limited^27^. However, even without the available technology, minimal documentation of the data is needed for implementing the appropriate technology. After a preliminary assessment, it became clear that a large proportion of the datasets used in this study were far from following the FAIR principles. The FAIR guiding principles were therefore reviewed individually and some were modified to better assess where the datasets fall on the FAIR continuum and not just if they meet the final objective. In particular, the principles related to “machine-actionability” were adjusted to mostly focus on “human-actionability”, meaning that the priority for our evaluation was to have data understandable by any person with no prior knowledge of the data.

In this study, the datasets were considered**:**

- **Findable**, when data and their metadata could be identified (have an identifier) unequivocally through time and were linked to each other. Despite the importance of the terms “globally unique” and “persistent” in the FAIR context, they were not included in our definition of the “Findable” principle. Indeed, being globally unique and persistent requires reliance on a third-party organisation that promises longevity and maintains these identifiers independently of the project/community^4^. Given the nature of the data included in our study, it was decided to only assess if there was a unique identifier, which may have been only relevant locally. The concept of “registered or indexed in a searchable resource” was not included in our assessment due to the absence of a recognised searchable resource for the agri-food business. Indeed, our datasets were not part of the research ecosystem, and if they can be considered as part of the agrifood community, we could not identify a standard community repository^4,28^. Note that the concept of “rich metadata” was covered only under the criteria “Interoperable” and “reusable”.
- **Accessible**, when access protocols to retrieve data and metadata were defined and documented. The concept of having “metadata accessible even when the data are no longer available” refers to the existence of persistent globally unique identifiers and was not included in the definition of ‘Accessible’ for the same reason as the one explained above.
- **Interoperable**, when data and metadata models were documented, and comprehensive vocabularies for naive human were used. In practice, this meant that to be considered ‘Interoperable’, a dataset should be accompanied by a document describing the dataset and its structure and (at least) a glossary defining the vocabulary used within the dataset. The concept of having vocabularies following FAIR principles was not included as this criterion was deemed too advanced for the datasets included in our study. The fact that (meta)data are meant to include qualified references to other (meta)data was intended to be assessed by looking at the metadata received as part of the survey. However, because too few metadata were shared by the survey respondents, this criterion was not included in the evaluation.
- **Reusable**, when a rich description of the data was available and documented. This also included provenance information (i.e., where are the data coming from, who collected them, how they are managed, including quality processes) and usage rights information (i.e., who owns the data, can they be used by others and if yes how and for what purpose (license)). Because of the very diverse nature of the data included in the study, the concept of having (meta)data aligned with domain-relevant community standards was not included in our evaluation. Indeed, our datasets could be considered as part of the agrifood community, as explained earlier, standards are unfortunately still lacking in this community^28^.

Twelve specific criteria were created to assess the compliance of study’s dataset to these adjusted FAIR principles based on the survey’s answers. The detail of the study datasets scoring scheme is presented in Table 2.

**Table 2:** FAIR compliance criteria and scoring scheme.

Compliance criteria	Compliance scoring
**FINDABLE criterion**	
**F1**. **Identifiable data**	1 = All elements to identify uniquely the data through time are available or a unique identifier is available.
	0.5 = One of the elements to uniquely identify the data is unavailable.
	0 = Not all previous conditions are fulfilled.
**F2**. **Identifiable metadata**	1 = All elements to identify uniquely the metadata through time are known or a unique identifier is available.
	0.5 = One of the elements defining the data’s metadata is unavailable.
	0 = Not all previous conditions are fulfilled.
**F3**. **Linked data and metadata**	1 = Metadata are updated automatically.
	0.5 = Metadata are updated manually.
	0 = No link or metadata not available.
**ACCESSIBLE criterion**	
**A1**. **Data access protocol**	1 = The data have a document describing their access protocols or have a URL.
	0 = Not all previous conditions are fulfilled.
**A2**. **Metadata access protocol**	1 = The data’s metadata have a document describing their access protocols or a URL.
	0 = Not all previous conditions are fulfilled.
**INTEROPERABLE criterion**	
**I1**. **Documented data structure**	1 = The data structure is documented.
	0 = Not all previous conditions are fulfilled.
**I2**. **Comprehensive vocabulary in the data**	1 = The vocabulary used was based on a standard, on a retrievable glossary or made collaboratively.
	0 = Not all previous conditions are fulfilled.
**I3**. **Documented metadata structure**	1 = The metadata’s structure is based on a standard schema or documented.
	0 = Not all previous conditions are fulfilled.
I**4**. **Comprehensive vocabulary in the metadata**	1 = The vocabulary used was based on a standard, on a retrievable glossary or made collaboratively.
	0 = Not all previous conditions are fulfilled.
**RESUABLE criterion**	
**R1**. **Available information about the data**	1 = All elements to construct or reconstruct the data’s metadata are available.
	0.5 = One element to construct or reconstruct the data’s metadata is missing.
	0 = Not all previous conditions are fulfilled.
**R2**. **Documented data collection and quality processes**	1 = Collection and quality processes are documented.
	0.5 = Some of the data processes are documented.
	0 = Not all previous conditions are fulfilled.
**R3**. **Data and metadata license**	1 = Data and its metadata are both licensed.
	0.5 = Only the data is licensed.
	0 = No license.

## Results

### Survey response rate

The survey response rate was 79% (responses received for 30 of the 38 datasets considered). The surveys were answered by 19 different respondents (r) from 11 organisations (4 commercial, 1 private non-commercial, 1 governmental and 5 public research organisations) but part of the same research consortium. About 26% of the respondents (r = 5) had only a clinical science background with no epidemiological or data science training. The others identified themselves as having a background in epidemiology (r = 12, 63%) and/or data science (r = 5, 26%).

Among the 19 respondents, only 32% (r = 6) reported having good knowledge of both FAIR and GDPR principles and having used them at least once. Almost half of the respondents (47%) have never applied GDPR principles, including three who did not know them. Similarly, 63% (r = 12) of respondents have never applied the FAIR principles, but only one did not know about them.

Furthermore, respondents were asked for consent to share the results of the survey (excluding personal information) with (i) the rest of the research project consortium, (ii) in a data repository for data reuse and (iii) in an anonymised format for publication. All respondents agreed that their answers could be used in a publication and published in an anonymised format. However, among the 30 survey answers^26^, 27% of the answers presented conditions (no sharing to anonymised sharing) before being shared with the rest of the research consortium despite privacy agreements (r = 8), and 37% of the survey answers didn’t receive consent to be deposited after anonymisation in a repository for data re-use by other scientists (r = 11).

### Quality of the answers

**Fig. 1:**
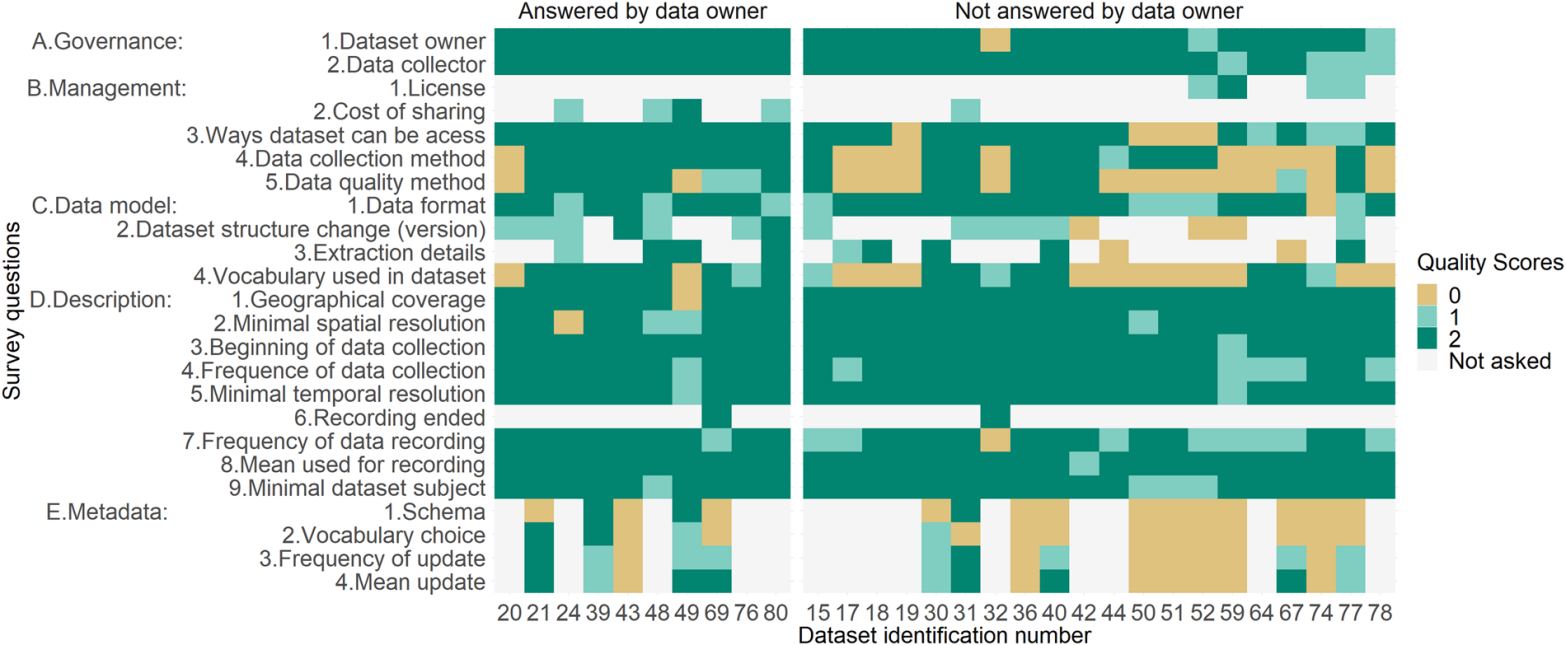
Quality of the answers.

The results of the assessment of the quality of the survey’s answers are presented in Fig. 1. The category ‘*A. Governance’* obtained the highest quality grade, with at least 87% of survey answers^26^ with a quality score of 2. Questions from the category ‘*D. Description’* also generally obtained answers of good quality (i.e., proportion of good answers (score = 2) above 75%), except for the question ‘*7. Frequency of data recording’ (i.e., only 66% of answers of good quality)*. The three remaining categories of the survey (i.e., ‘*B. Management’, ‘C. Data model’* and *‘E. Metadata’)* obtained poor-quality grades (i.e., rate of good answers (score = 2) between 12 and 70%). The only exception was the question related to *‘3. ways dataset can be accessed’* (i.e., included in category ‘*B. Management’*), where 77% of the answers got a good quality score.

### Characterisation of study datasets

#### Overview

The number of datasets (n) available per species was similar for swine (n = 10, 33%), salmonids (n = 9, 30%), and cattle (n = 9, 30%), including two datasets with both swine and cattle data (7%). Poultry was the least represented species (n = 4, 13%). The datasets covered 11 different European countries. Most datasets were not in English (n = 20, 67%), and one was translated. The main information collected in these datasets were laboratory results (n = 16, 53%), biosecurity information (n = 15, 50%), production results (n = 14, 47%), treatment information (n = 13, 43%) and clinical observation (n = 13, 43%). Other collected data (animal movements, economic information and behaviour observation) were collected in 6 or fewer datasets, and none of the datasets contained data collected through sensors. Most datasets contained data collected for more than one purpose (n = 19, 63%). Still, all were created to answer operational needs such as the collection of production information (n = 17, 57%), support surveillance (n = 16, 53%), ensure compliance with legislation (n = 11, 37%), diagnostic laboratory activities (n = 8, 27%), or other activity such as business management or quality control (n = 4, 13%). While none of the original data was collected primarily for research, three datasets were created for research purposes through the integration of different data sources.

#### Data collection process

Respondents did not provide detailed documentation about the data collection processes for most of the datasets. However, based on the surveys’ answers, 6 datasets integrated information from different types of stakeholders (e.g., laboratories and farmers or slaughterhouses and veterinary clinics). The other datasets either collected information from a single source (n = 11) or multiple stakeholders of the same type (n = 12) (e.g., only laboratories or only integrators). Data was mostly collected using an automated approach such as an application programming interface (API) or digital forms (n = 18). The remaining datasets were either collected manually (n = 6), or the data collection approach was not reported (n = 6).

#### Data quality

Half of the datasets had some form of documentation related to the data quality processes implemented (n = 15). Most of this documentation was not provided to the authors, but some information was provided in the open questions of the research and documentation. Based on this information, six types of data quality processes were identified and reported as used in the study datasets:

- the use of closed formats in the data collection process (n = 14),
- training of the data collectors (n = 5),
- manual checks of the collected data (n = 5),
- automated quality checks of the collected data (n = 14),
- inspection and verification of the data quality of the dataset (n = 4),
- feedback loops (sharing information on the dataset to the data producer (n = 17) and
- the use of standards and procedures such as laboratory quality schemes (n = 18).

#### Data model

The data model was documented for most of the study’s datasets (n = 25); details are available in Table S1 (see supplementary xlsx file). Datasets were mainly stored in tabular formats, allowing for the structuration of the content in a tabular format, either as spreadsheets (8 in collections of multiple spreadsheets and 7 in single spreadsheets) or as relational databases (n = 14). Only one dataset was stored as multiple PDFs.

Most datasets have been modified since their creation (n = 16). However, information about what had changed was available for only 9 of them. However, for a third of the study’s datasets, the available datasets are extractions (n = 11).

None of the datasets was reported using an identified, retrievable, and standardised vocabulary. However, five types of practice linked to vocabulary were identified based on the respondent’s answer and complementary research: ‘Data glossary available to the authors’ (n = 8), ‘Reported use of a vocabulary standard unidentifiable by the authors’ (n = 2), ‘Vocabulary co-defined in collaboration between multiple stakeholders’ (n = 4) and ‘Vocabulary designed and defined by the data owner’ (n = 12). This information was unavailable for 6 of the datasets.

#### Metadata

Out of all the datasets, almost half of them didn’t have identified metadata available (n = 14). Among the remaining 16 datasets, only one was structured in a standardised schema, linked data and automatic updates, allowing for machine readability. For the other fifteen datasets, even if metadata existed, it has been difficult to get detailed information about them. Indeed, schema structure, vocabulary choice and update frequency were often empty or reported as unknown by the respondents. Details per dataset are available in Table S1 (see supplementary xlsx file).

### GDPR accountability

**Fig. 2:**
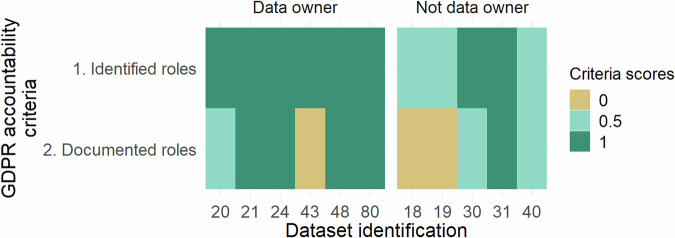
GDPR accountability scoring for the eleven study’s datasets, including personal data.

Based on the survey’s answers^26^, only eleven of the datasets gathered personal data. The results of the GDPR accountability evaluation for these eleven datasets are detailed in Fig. 2. No differences in GDPR scores between the species were investigated because of the small number of datasets evaluated.

The data controller could not be identified for three of the datasets. The role of the data controller and a clear identification of the data subject (as defined in the GDPR definition) were both documented in less than half of the evaluated datasets (n = 5). In this survey, the GDPR accountability scores are higher when the data owner completed the surveys (mean score of 1.75) than responses from data users (mean score of 1.1).

### FAIR compliance

**Fig. 3:**
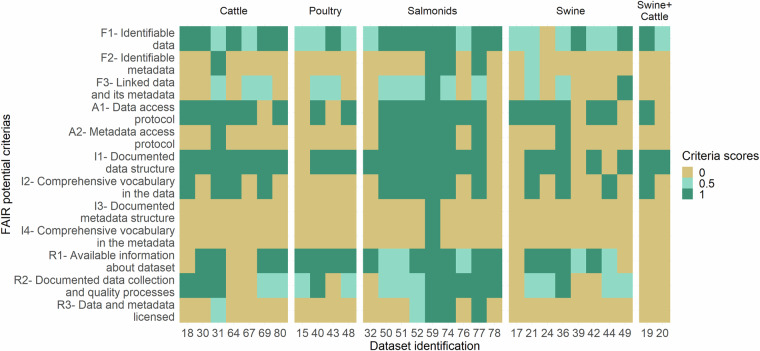
FAIR compliance scores for the study’s datasets organised by species.

The FAIR compliance scores were low for most of the datasets (Fig. 3). Four datasets fulfilled at least half of study’s FAIR compliance criteria, and only one fulfilled all. Three of these four datasets were publicly available and contained information about salmonids.

The FAIR compliance scores varied depending on the FAIR principle studied:

- **Findable**: Sixteen of the thirty datasets could be clearly identified over time (F1), and four had fully identifiable metadata (F2). The link between the data and the metadata was, in most cases (n = 28), not clearly made (F3).
- **Accessible**: Twenty-two of the thirty datasets had documented processes to render the data accessible to others (A1), but only eight were associated with metadata access protocols (A2).
- **Interoperable**: Twenty-five datasets had a documented data structure (I1), and about half of the datasets (n = 14) reported using a vocabulary that should be understandable by someone having no prior knowledge of the data (I2). For the metadata, one dataset had a documented metadata structure (I3) and used a standardised vocabulary in its metadata (I4).
- **Reusable**: 18 datasets had the information required to construct rich metadata (R1), even if this information was not necessarily formalised in existing metadata (F2). One-third of the datasets (n = 10) had full documentation on the data collection and quality processes used (R2). Very few study’s (meta)data (n = 3) appeared to be protected by a licence (R3).

## Discussion

This study assessed how 30 datasets^26^ produced by stakeholders from four major European livestock production chains can be reused in a research framework. Our main result is that getting a full and standardised data description was challenging. Often, information was missing and/or not reported by the respondents. This was similar for all species. For example, almost half of the datasets had no identified metadata available (defined as all elements describing the data^6^). When metadata existed, their structure and the number of details varied greatly between datasets. This lack of good data description is illustrated by the low scores obtained by the datasets for the FAIR compliance score related to metadata. The metadata of the data included in this study were rarely findable, accessible, and/or interoperable. None of the datasets included in this study used predefined metadata schema such as DCAT (https://www.w3.org/TR/2024/CR-vocab-dcat-3-20240118/) nor any of the data formats developed specifically to address the issue of syntactic interoperability in epidemiological data^23,29^. The absence of documented metadata indicates that the existing metadata standards are not broadly embraced for this type of data but, even more importantly, that the transfer of knowledge about datasets remains mostly based on direct interpersonal communication and not on documentation. This is a major issue not only in terms of data interoperability but also in terms of increased risk of loss of information and analytical errors due to misinterpretations and critical inefficiencies. In practice, it means that the data are often not directly usable by a “naïve data user” unless there is direct and extensive communication with “someone” who already knows the data. It should not only be seen as a problem for researchers who may want to reuse these ‘non-research’ data in a research framework, but it should also be a concern for anyone intending to use these data for decision-making.

Similarly, vocabulary standards and data glossaries were rarely used, and half of the datasets had no comprehensive vocabulary, making them poorly interoperable from a FAIR perspective. Several ontologies or standard vocabularies have been developed in the animal health sector including FAANG for the animal genome^30^, FoodOn for the food chain^31^, AGROVOC for agricultural concepts (https://agrovoc.fao.org/browse/agrovoc/en/index); ATOL, EOL and AHOL for livestock traits, environment and health (https://www.umrh.inrae.fr/ontologies/visualisation/public/); and AHSO on animal health surveillance^22^. None of the existing standards is perfect. For example, existing ontologies are not complete, and more work would be needed to link them together^22,28^. The structured vocabulary AGROVOC provides translation for some of the terms in multiple languages but does not confront the different meanings associated with each translation or within a language, which can be confusing. As an illustration, heifers are defined in AGROVOC as both “Cows that have not yet borne a calf” or as “female cows that have not yet born two calves”^32^. Future work should address some of the existing vocabulary standards’ limitations and better promote their broad use when a new dataset is created.

Aside from the lack of content description, our study also highlighted the lack of information available on the quality and flaws of the data. The reported processes to ensure data quality were diverse and not always documented in detail. The absence of (or lack of access to) appropriate documentation describing the dataset flaws has been identified as a major issue for data (re)usability and interoperability^5^. To be able to report data quality, people must have access to clear guidance on how to assess and report data quality^33^. Data quality standards have been developed for specific domains (e.g., biodiversity data^34^) but to our knowledge, not for animal health data, even if several studies have assessed in more or less formal ways the quality of animal health data^35–38^. Future studies should build on existing work to develop data quality assessment standards relevant to the specific field of animal health.

Despite the regulatory framework in place, only five out of the eleven datasets with personal data fulfilled all the criteria used to assess their GDPR’s accountability principle’s compliance. This apparent low level of compliance with GDPR in terms of accountability could be explained by the reported low familiarity of the respondents with GDPR. Indeed, the respondents were often unfamiliar with the definitions of ‘personal data’, ‘data controller’, and ‘data subject’, which may have caused errors in the answers provided to the survey. In this context, the real level of compliance of the datasets with GDPR remains unknown. The difference in score depending on the status of the survey respondents (i.e., data owner or simple data user) suggests that a loss of information on data governance may occur during the data sharing process and/or that data users lack empowerment on this topic. It is unknown if the respondents, who were just data users, did not have access to the information because they didn’t ask for it or could not access it. Either way, the GDPR indicates that data governance roles are supposed to be clear and available to all, including data users. There is thus a need to improve the training of people working with data, including researchers, about the requirements of the GDPR as soon as personal data are involved^12,15,20,39^. addressing agricultural data privacy reality^10,21^.

One of the limitations of this study was that it was not always clear whether the information did not exist or was just not available or known to the survey respondents. In addition, when the information existed and was available, it may not have always been understood by the survey respondents, as suggested by the overall low-quality score of the surveys. This hypothesis was further confirmed when we tried to complete the survey answers with information coming from other sources. These *a posteriori* edits of the survey’s answers may have artificially changed the scores associated with some of the datasets. Still, more importantly, they highlighted discrepancies between the survey answers and the reality. One of the best examples is the publicly available dataset n°59, which was the only dataset which used standard vocabulary and had machine-readable metadata (i.e., XML and RDF format available). However, the survey respondent indicated that the dataset didn’t have associated metadata and was only available in a poorly interoperable format (i.e., pdf). This error is likely due to a lack of technical knowledge of the survey respondent, who was probably unable to understand the information in machine-actionable data format. The use of a questionnaire survey over direct data access has likely biased the results of this study but also highlighted the need to increase the overall skills of the animal health scientist’s community in data management good practices, GDPR and FAIR compliance. This is perhaps the most important finding of our study: the overall poor quality of the data collected and lack of understanding of some key concepts by the survey respondents are a good illustration of the challenges ahead to improve data reuse in the veterinary epidemiology domain.

Even if the assessment of datasets’ compliance with the FAIR principles is the heart of this study, ironically, the data collected in this work do not themselves follow those principles. Indeed, a large proportion of the respondents (37%) did not agree to make their answers publicly available even after anonymisation. Therefore, the detailed outputs of the surveys cannot be deposited in a repository. Similarly, a non-negligible proportion of survey respondents did not want to share their un-anonymized answers to the survey or the metadata of their datasets with other researchers from the same research project despite specific data-sharing agreements signed at the project level. If protection of the data themselves might be legitimate, especially when referring to non-scholarly data, protection of the metadata is more questionable. One option is that the project partners who were not the owners of the data were maybe unsure if they were allowed or not to share this information. In that instance, it is possible they prefer to go for the “safer” option to avoid any risk of losing the trust of their data provider, especially when data was obtained through interpersonal relationships. Another possibility is that, because of the absence of documents describing the data (i.e., the metadata), project partners may not have been feeling comfortable sharing information, which was somehow not validated. This discomfort could also fuel the researcher’s fear of being accused of flawed interpretation or falsification, which were both identified as a key barrier to academic data sharing^40^ Future work should investigate the reluctance of stakeholders to share their metadata, when they were available, and understand whether this is related to legitimate fears of sharing sensitive unvalidated information or rather to misunderstanding of some concepts such as “data privacy” or “personal data”. Indeed, our study pointed out the fact that some critical knowledge in these domains might be missing at the community level and that more systematic training should be implemented and/or new guidelines should be developed.

This study assessed compliance with the FAIR and GDPR principles. However, as highlighted already in the Introduction, the current guidelines are not really fit for purpose for the type of data considered in this study: non-scholarly data intended to be used for research. Developing data management guidelines that are more adapted to the specific nature of these data could help improve their reusability for research purposes, and strengthen disease control and surveillance. However, beyond the guidelines, there is also a need to identify and/or develop tools and technology that could be used as references or standards by the animal health community. Indeed, in our study, several FAIR criteria were modified or not assessed because of the absence of such standards for this specific community. For example, DataCite (https://datacite.org/) or Crossref (https://www.crossref.org/) are creating and sustaining globally unique and persistent identifiers for the data of the research community. However, such organisations do not yet exist for non-scholarly data. Defining and implementing these standards might take some time as animal health is a multifaceted domain at the interface of different communities (e.g., nutrition, behaviour, genetics, environment, etc.) and stakeholders (e.g., government, private industry, researchers, etc.), which may not have standards defined either, nor be linked. Furthermore, it should be noted that the objectives defined by the FAIR principles may never be fully attained as the stakeholders producing the data may not have the resources or the need to follow those guidelines fully^28^. Future studies could focus on better describing the level of FAIR maturity non-scholarly datasets should be expected to reach to sustainably support data reusability in the animal health domain.

Additionally, assessing if these specific data are properly managed for their multiple purposes may require using other criteria. For example, the Global Burden Of Animal Diseases project (GBADs) underlined the need to include the principle of “Security” (FAIRS data) when integrating and reusing data from multiple sources^41^. Similarly, Dórea, F. *et al*. promoted the idea of FAIR-ER data, extending the FAIR principles to the concepts of “Extendibility” and ‘Reproducibility”^42^. Considering the extendibility of data is particularly relevant when integrating different sources of data and/or when considering continuous data collection (e.g., data collected for disease surveillance). In this context, tracking data provenance is critical. Similarly, data reproducibility should be considered when multiple data curation steps might be involved in building a dataset. More work would be needed to make the FAIR guiding principle more adapted to the specificities of the data used in animal health.

Our study highlighted major gaps in terms of (i) compliance with the accountability principle (identification of data governance roles) and (ii) implementation of good data management practices, especially in terms of the use of metadata and standard vocabularies. Both are possibly related to an overall lack of knowledge of data management, good practices, and regulations and how to implement them. Previous studies have already shown that the specific skills required to understand and handle machine-readable standards are not broadly available in the epidemiology community yet^15^, and may even constitute a barrier to data sharing for both funders and scientists^2,9,43^. Our study highlights that even simpler concepts are not always well understood or implemented, highlighting critical gaps in terms of skill set. As already highlighted by others^15^, more systematic data management training is needed in graduate and continuous education programmes, to develop awareness around concepts such as the FAIR principles or GDPR and to improve the basic skills of epidemiologists and animal health professionals in data management good practices. To be efficient, this training would have to highlight the benefits of good data management as it can appear not just complicated but also timely and costly for individual researchers. Indeed, adhering to any particular standards requires implementation efforts that should not be underestimated. The development of more accessible guidelines and *ad hoc* training may also be options to reach a large number of people quickly and rapidly raise the competencies of current and next generation of scientists operating in the animal health community, which is an essential requirement to reap the full benefits offered by the rapidly growing volume of heterogeneous data available in livestock productions systems.

## Supplementary information

Full survey

Oversized Supplementary Table 1

## Data Availability

The primary answers to the online survey supporting this study’s findings cannot be publicly available due to the absence of consent from several study participants. However, the anonymised and processed data are all available in figshare repository at Delavenne *et al*.^26^, which also contains for each variable the link to the original survey question and the process used to obtain the result. Please note that the supplementary table available as part of this paper provides information to link the results of the study with the anonymized survey responses and may assist in explaining some of the column values.

## Code availability

No specific custom code has been used to conduct this descriptive study. Therefore, the code used to generate the results presented is not made available.
