# Supplementary material for: Reusability challenges of livestock production data to improve animal health
Source: Sci Data. 2025 Mar 19;12:458. doi: 10.1038/s41597-025-04785-4 (PMC11923216; doi:10.1038/s41597-025-04785-4)
Supplement: Supplementary file 1 — Full survey [file 41597_2025_4785_MOESM1_ESM.pdf]

Dear data owners,

Thank you for your willingness to participate in DECIDE research project and to answer this survey.

DECIDE's goal is to develop data-driven decision support tools to improve the surveillance and control of non-EU-regulated contagious animal diseases. To meet this objective some data needs to be shared between data owners and researchers to be able to develop the tools. Then we need to understand how these tools could be used on other data available in the project.

Combining and comparing data from multiple sources comes with challenges in terms of data privacy, security, and integration.

To identify the potential data that can be shared and used within the DECIDE consortium, create further data sharing opportunities, the leaders of WP1 designed this survey for data owners to describe their datasets. Hence one questionnaire should be answered per dataset. In this survey, a dataset is defined as *a single structure (CSV file, relational database, etc..) managed by a single entity (unique data owner, data controller, and custodian),* with the data owner being *the legal entity who has the right to grant access to or to share the dataset (containing or not personal data) under its control.*

Examples:

For example, I have multiple excel files handed to me by two different people from the same laboratory containing information about lab results and necropsy per farm. In some cases, those files are linked to each other because, after the necropsy investigation, samples were taken to explore antimicrobial presence. Therefore, despite being made of multiple files, we would be considered them as a unique dataset for this questionnaire because coding variables have been harmonized among the files, there is a similar dataset structure across the files and the files have been managed by one entity (the same lab). A unique questionnaire should be answered and not one per file. A relational database is a collection of data stored and accessed electronically and organized in a relational model. Data are organized in more than one table of columns and rows, with a unique key identifying each row and logical connections between each table. Therefore a unique questionnaire should be answered, and not one per table. On a governmental server through an application programming interface, veterinary services of a country can import information about herd status and lab analysis into a relational database and they do so to manage animal health and undergo surveillance activities. However, on this same server, the ministry of agriculture also conducts a yearly inquiry, asking for each region to answer general aggregated questions concerning the general animal health status. Despite being hosted and accessed similarly, those two datasets are constructed differently with no link between them despite concerning similar topics

Section A: Respondent profile

A1. Could you specify your name and contact information?

The personal information we collect from you is only meant to be used by DECIDE WP1 teams for complementary information if needed and to send you feedback. For the analaysis, all personnal information will be anonymised.

First name ☐

Surname ☐

Email address ☐

A2. Can we use this email address, if we needed to clarify some of your answers and to provide you with complementary information about this questionnaire?

- Yes ☐
- No ☐

A3. Please check the box or boxes that best describe your academic background

- Animal husbandery ☐
- Biology ☐
- Computer science ☐
- Data science ☐
- Epidemiology ☐
- Medical doctor ☐
- Sociology ☐
- Veterinary science ☐
- Other ☐

Other

☐

A4. **Please choose the variable that best describes your employer's category**

- Governmental authority ☐
- Inter-governmental organization ☐
- Governmental organization ☐
- Academia (such as university, research institute) ☐
- Private sector ☐
- Non-governmental organization ☐
- Other ☐

Other

[ ]

A5. **Who is your employer or your institutional affiliation?**

[ ]

A6. **How familiar are you with those concepts?**

	I don't know those concepts	I have heard about them	I have had to tackle those concepts once	I have applied those concepts multiple times in the different context
GDPR legislation	☐	☐	☐	☐
FAIR principles	☐	☐	☐	☐

A7. **What is your role or employer's role to the dataset?**

- Data subject - Person whose data is processed (a client, a farmer, etc...) in a dataset. ☐
- Data collector - Legal entity who is responsible for creating or capturing data. ☐
- Data owner - Legal entity who has the right to grant access to or to share certain personal or non-personal data under its control ☐
- Data user - Legal entity who has lawful access to the dataset and is authorized to use that data for commercial or non-commercial purposes. ☐

A8. **You confirmed that you or your organization is the data owner of the dataset, as a person, do you have any of those two roles?**

- Data controller – The person who decides why and how personal data will be processed. If you’re an employee in your organization who handles data and knows how to use it, this is you. ☐
- Data custodian – The person who manages the actual data. This role manages servers, backups, or networks. It may provide access per the data owner’s rules and has mastery of a data schema and lineage. This role knows exactly where data is located but does not know how to correctly use it. ☐

Section B: Dataset's profile - basic information

B1. Was the dataset already identified in the previous WP1 inventory?

If you are not sure, please answer no.

Yes ☐
No ☐

B2. What is the dataset's name in the inventory?

B3. What is the dataset's name?

B4. What is the dataset's thematic area?

	Yes	Uncertain	No
Animal movement data (between different locations)	☐	☐	☐
Farm data (including biosecurity and barn environment)	☐	☐	☐
Animal sensor data (from the machine making automated measures such as milking robots, automatic thermometers, camera's, or others)	☐	☐	☐
Laboratory results data (such as pathology, bacteriology, PCR…)	☐	☐	☐
Clinical data (such as symptoms, diseases...)	☐	☐	☐
Treatment data (such as antimicrobials, anthelminthic, sealice treatment...)	☐	☐	☐
Production data (including mortality, reproduction, growth, feed intake, meat quality)	☐	☐	☐
Economic data (cost, prize, revenue…)	☐	☐	☐
Welfare data (including behaviour data)	☐	☐	☐

B5. Please choose which DECIDE species is recorded in the dataset

Cattle ☐
Poultry ☐
Porcine ☐
Salmonids ☐

B6. **Briefly describe the dataset**

B7. **What language is used in the dataset?**

- Albanian ☐
- Arabi ☐
- Armenian ☐
- Azerbaijani ☐
- Basque ☐
- Belarusian ☐
- Bengali ☐
- Bosnian ☐
- Breton ☐
- Bulgarian ☐
- Catalan ☐
- Chinese ☐
- Corsican ☐
- Croatian ☐
- Czech ☐
- Danish ☐
- Dhivehi ☐
- Dutch ☐
- English ☐
- Esperanto ☐
- Estonian ☐
- Faroese ☐
- Finnish ☐
- Flemish ☐

- [ ] French
- [ ] Frisian
- [ ] Galician
- [ ] Georgian
- [ ] German
- [ ] Greek
- [ ] Greenlandic
- [ ] Hebrew
- [ ] Hindi
- [ ] Hungarian
- [ ] Icelandic
- [ ] Indonesian
- [ ] Interlingua
- [ ] Irish
- [ ] Italian
- [ ] Japanese
- [ ] Kashubian
- [ ] Kazakh
- [ ] Khmer
- [ ] Korean
- [ ] Kurdish
- [ ] Kyrgyz
- [ ] Lao
- [ ] Latin
- [ ] Latvian
- [ ] Lithuanian
- [ ] Luxembourgish
- [ ] Macedonian
- [ ] Malagasy

Malay ☐
Maltese ☐
Minangkabau ☐
Moldavian ☐
Mongolian ☐
Montenegrin ☐
Nepali ☐
Northern Sami ☐
Norwegian ☐
Occitan ☐
Ossetian ☐
Persian ☐
Polish ☐
Portuguese ☐
Romani ☐
Romanian ☐
Romansh ☐
Russian ☐
Scottish Gaelic ☐
Serbian ☐
Serbo-Croatian ☐
Sinhala ☐
Slovak ☐
Slovenian ☐
Spanish ☐
Swahili ☐
Swedish ☐
Tajiks ☐
Tamil ☐

Thai ☐

Tibetan ☐

Turkish ☐

Turkmen ☐

Ukrainian ☐

Urdu ☐

Uzbek ☐

Valencian ☐

Vietnamese ☐

Walloon ☐

Welsh ☐

Yiddish ☐

Zulu ☐

B8. What is the dataset's intended purpose?

Monitoring production ☐

Research ☐

Surveillance ☐

Legislation compliance ☐

Laboratory results ☐

Bookkeeping ☐

Product quality ☐

B9. Has this dataset been used for published research or published reports?

Yes ☐

No ☐

B10. What is the reference of the published research (DOI) or where are the reports accessible (URL)?

1 ☐☐☐☐☐☐☐☐☐☐

2 ☐☐☐☐☐☐☐☐☐☐

3 ☐☐☐☐☐☐☐☐☐☐

4 ☐☐☐☐☐☐☐☐☐☐

Section C: Dataset's profile - governance

C1. Is there an existing written document identifying who is the owner of the dataset?

Data owner - Legal entity who has the right to grant access to or to share certain personal or non-personal data under its control

Yes ☐

No ☐

C2. Can you identify the dataset owner?

Data owner - *Legal entity who has the right to grant access to or to share certain personal or non-personal data under its control*

Yes ☐

No ☐

C3. Who is the dataset owner?

Data owner - *Legal entity who has the right to grant access to or to share certain personal or non-personal data under its control*

C4. Is there an existing written document identifying who is the dataset collector?

Data Collector - Legal entity who is responsible for creating or capturing data.

Yes ☐

No ☐

C5. Can you identify the dataset collector?

Data Collector - Legal entity who is responsible for creating or capturing data.

Yes ☐

No ☐

C6. Who is the dataset collector?

Data Collector - Legal entity who is responsible for creating or capturing data.

C7. Does the dataset contain personal data?

Personal data - Any information that relates to an individual who can be directly or indirectly identified.

Hence, names and email addresses are personal data. Location information, ethnicity, gender, biometric data, religious beliefs, web cookies, and political opinions can also be personal data. Pseudonymous data can also fall under the definition if it's relatively easy to ID someone from it. (GDPR definition)

Yes ☐

No ☐

C8. Is there an existing written document identifying who is the dataset's subject?

Data subject - Person whose data is processed (a client, a farmer, etc...) in a dataset.

Yes ☐

No ☐

C9. What were the conditions under which the personal data were collected?

Data collected by public authorities for its missions

Comment

Data collected through unambiguous consent - the data subject are aware of who can use their information, for which purpose, and they know how they can remove easily their consent and retrieve their information.

Comment

Data collected under clear contract

Comment

C10. Is there an existing written document identifying who is the dataset controller?

Data controller – The person who decides why and how personal data will be processed in the company or organization that owns the data.

Yes ☐

No ☐

C11. Can you identify the dataset's controller?

Data controller – The person who decides why and how personal data will be processed in the company or organization that owns the data.

Yes ☐

No ☐

C12. Is the dataset's controller part of the same organization as the data owner *(data management insourced and not outsourced)*?

Data controller – The person who decides why and how personal data will be processed in the company or organization that owns the data.

Yes ☐

No ☐

C13. Is there an existing document identifying who is the dataset custodian?

Data custodian – The person who manages the actual data. This role manages servers, backups, or networks. It may provide access per the data owner's rules and has mastery of a data schema and lineage. This role knows exactly where data is located but does not know how to correctly use it.

Yes ☐

No ☐

C14. Can you identify the dataset's custodian?

Data custodian – The person who manages the actual data. This role manages servers, backups, or networks. It may provide access per the data owner's rules and has mastery of a data schema and lineage. This role knows exactly where data is located but does not know how to correctly use it.

Yes ☐

No ☐

C15. Is the dataset's custodian part of the same organization as the data owner *(data management insourced and not outsourced)*?

Data custodian – The person who manages the actual data. This role manages servers, backups, or networks. It may provide access per the data owner's rules and has mastery of a data schema and lineage. This role knows exactly where data is located but does not know how to correctly use it.

Yes ☐

No ☐

Section D: Dataset's profile - management

D1. Is the data set protected by a specific license such as creative commons?

Yes ☐

No ☐

D2. What is the dataset license?

D3. Is there an existing written document defining how the data can be accessed and by who?

Yes ☐

No ☐

D4. **Where is the dataset hosted?**

- On a single computer ☐
- On a server ☐
- On a cloud ☐

D5. **What is the general dataset access right?**

- Non-public - autorisation requiered ☐
- Public - No limitations ☐
- Restricted - registration required ☐

D6. **Where is the dataset accessible (URL)?**

D7. **Is there a written document(s) describing the methodology used during data collection to guarantee data completeness and provenance?**

- Yes ☐
- No ☐

D8. **Can you share this document?**

- Yes ☐
- No ☐

D9. **Can you quickly describe this method?**

D10. **Is there a written document describing the methodology to guarantee data quality?**

- Yes ☐
- No ☐

D11. **Can you share this document?**

- Yes ☐
- No ☐

D12. **Can you quickly describe this method?**

D13. **Have you identified costs associated with data sharing?**

Yes ☐

No ☐

D14. **Can you give us a broad estimate of the data sharing cost in euro?**

Section E: Dataset's profile - structure

E1. **Is the dataset's structure documented?**

Yes ☐

No ☐

E2. **How is the dataset formatted? Specify the media type (csv, xml, SQL, ...)**

Unique spreadsheet (csv, Excel file, ...) ☐

Multiple spreadsheet (csv, Excel file,...) ☐

Relational database ☐

Other ☐

E3. **Has the structure of the dataset (number of tables, columns, variables, language used, ...) changed since its original creation?**

Yes ☐

No ☐

E4. How many times did the dataset structure change since its creation?

What is the number of the current version?

E5. Is the dataset an extraction of a larger one?

Yes ☐

No ☐

E6. When did the extraction occur?

E7. Was the dataset constructed using a standard vocabulary/ontology?

*A **standard vocabulary** is a list of words that have been defined, published, and shared with a community to allow for unambiguous identification of the concept they stand for. Standard vocabularies can have different formats including ontologies. An **Ontology** can be roughly described as a vocabulary with hierarchies, meaningful relations among concepts, and their constraints.*

Yes ☐

No ☐

E8. You indicated that the dataset was fully or partially based on a standard ontology/vocabulary.

*A **standard vocabulary** is a list of words that have been defined, published, and shared with a community to allow for unambiguous identification of the concept they stand for. Standard vocabularies can have different formats including ontologies. An **Ontology** can be roughly described as a vocabulary with hierarchies, meaningful relations among concepts, and their constraints.*

What is the standard ontology/vocabulary's name?

Where is it accessible?

How much of the dataset is based on it?

E9. How was the vocabulary in the dataset defined?

E10. Do you know if your dataset uses a language that allows knowledge representation such as owl or rdf?

Yes ☐

No ☐

E11. What is the language used?

Section F: Dataset's profile - subject

F1. Choose the most appropriate spatial coverage of the dataset and specify if needed?

For example, if data cover a European country, specify which one.

- Europe ☐
- Multiple European countries ☐
- A European country ☐
- Multiple regions of a european country ☐
- A region ☐
- Other ☐

F2. What is the spatial resolution of the data set (smallest unit)?

- Exact coordinates ☐
- ZIP code ☐
- NUTS 1 ☐
- NUTS 2 ☐
- NUTS 3 ☐
- Adresse ☐
- Commune ☐
- Other ☐

Other

F3. **What coordinate reference system is used in the dataset (please specify the EPSG code)?**

F4. **When did the data collection begin, indicating the year?**

Please only enter the year using the following format 'yyyy'.

F5. **Is the data collection still ongoing?**

Yes ☐

No ☐

F6. How frequently is the data collected?

- Annual ☐
- Biennial ☐
- Bimonthly ☐
- Biweekly ☐
- Continuous ☐
- Daily ☐
- Irregular ☐
- Monthly ☐
- Quaterly ☐
- Semiannual ☐
- Semimonthly ☐
- Semiweekly ☐
- Three times a month ☐
- Three times a week ☐
- Three times a year ☐
- Triennal ☐
- Twice a day ☐
- Unknown ☐
- Weekly ☐
- Other ☐

Other

F7. How frequently is the collected data updated into the dataset?

- Instantly as it is collected ☐
- Annual ☐
- Biennial ☐
- Bimonthly ☐
- Biweekly ☐
- Daily ☐
- Irregular ☐
- Monthly ☐
- Quaterly ☐
- Semiannual ☐
- Semimonthly ☐
- Semiweekly ☐
- Three times a month ☐
- Three times a week ☐
- Three times a year ☐
- Triennal ☐
- Twice a day ☐
- Unknown ☐
- Weekly ☐
- Other ☐

Other

F8. **What is the dataset temporal resolution (smallest unit)?**

- Second ☐
- Minute ☐
- Hour ☐
- Date ☐
- Month ☐
- Year ☐
- Season ☐
- Production batch ☐
- Growth stage ☐
- Other ☐

Other

F9. **When did the data collection end, indicating the year?**

Please only enter the year using the following format 'yyyy'.

F10. **The activities in the dataset have been recorded by what kind of agents? If it is a mix, please define a bit more in the "other category".**

- Human ☐
- Sensors (or machine for a laboratory for exemple) ☐
- Unknown (dataset is the result of an aggregation activity of other data) ☐
- Other ☐

Other

F11. What is the dataset's subject (smallest unit)? If possible give its definition.

- Sample
- Animal
- Animal batch
- Herd
- Farm
- Other

Section G: Dataset's profile - metadata

G1. Are some or all of the previous information answered about the dataset's profile documented?

Do you have documented metadata?

- Yes
- No

G2. Was that information formatted following a model/ metadata schema?

Labeling, tagging, or coding system used for recording cataloging information or structuring descriptive records...

- Yes
- No

G3. Is the used metadata schema/model a standard one?

- Yes
- No

G4. You indicated that the metadata schema was based on a standard.

How is it name?

Where is it accessible?

G5. **Is there a written document describing how the metadata schema was constructed?**

Yes ☐

No ☐

G6. **Was the dataset's metadata fully or partially encoded using a standard vocabulary/ontology?**

*A **standard vocabulary** is a list of words that have been defined, published, and shared with a community to allow for unambiguous identification of the concept they stand for. Standard vocabularies can have different formats including ontologies. An **Ontology** can be roughly described as a vocabulary with hierarchies, meaningful relations among concepts, and their constraints.*

Yes ☐

No ☐

G7. **You indicated that the dataset's metadata was based on a standard ontology or vocabulary.**

*A **standard vocabulary** is a list of words that have been defined, published, and shared with a community to allow for unambiguous identification of the concept they stand for. Standard vocabularies can have different formats including ontologies. An **Ontology** can be roughly described as a vocabulary with hierarchies, meaningful relations among concepts, and their constraints.*

What is the standard ontology/vocabulary's name? ____________

Where is it accessible? ____________

How much of the dataset is based on it? ____________

G8. **How was the vocabulary in the dataset's metadata defined?**

G9. **Do you know if your dataset uses a language that allows knowledge representation such as owl or rdf?**

Yes ☐

No ☐

G10. **What is the language used?**

G11. How often is the dataset's metadata updated?

- Annual ☐
- Biennial ☐
- Bimonthly ☐
- Biweekly ☐
- Continuous ☐
- Daily ☐
- Irregular ☐
- Monthly ☐
- Quaterly ☐
- Semiannual ☐
- Semimonthly ☐
- Semiweekly ☐
- Three times a month ☐
- Three times a week ☐
- Three times a year ☐
- Triennal ☐
- Twice a day ☐
- Unknown ☐
- Weekly ☐
- Other ☐

Other

G12. **How are those datasets' metadata updated?**

- Automatically ☐
- Manually ☐
- Other ☐

Other

G13. **Is the process of updating the dataset's metadata documented?**

- Yes ☐
- No ☐

G14. **Is there a procedure to define access to the dataset's metadata?**

- Yes ☐
- No ☐

G15. **Is the dataset's metadata protected under a license?**

- Yes ☐
- No ☐

Section H: Sharing the datasets

H1. **Are you part of DECIDE consortium?**

- Yes ☐
- No ☐

H2. **Has the dataset already been shared with one of DECIDE's partners (other than the owner)?**

- Yes ☐
- No ☐

H3. **When was the dataset shared with the partner?**

H4. **What was shared?**

- Full dataset ☐
- Copy of the dataset ☐
- Unaggregated extraction of the data set ☐
- Aggregated extraction of the data set ☐
- Other ☐

Other

H5. **How was the dataset shared?**

- By a single download (via url, mail, hardware) ☐
- Through a specific service such as an API ☐
- Direct access to dataset's host (server, cloud, other) ☐
- Other ☐

Other

H6. **Was the metadata shared with the dataset?**

- Yes ☐
- No ☐

H7. **Can the dataset and its metadata be shared by other members of the consortium under the DECIDE joint-controller agreement?**

- Yes ☐
- Maybe, if (anonymization, just some variables, etc)? ☐
- No, why? ☐

H8. **Can the dataset and its metadata be reused by another researcher outside of DECIDE consortium?**

- [ ] Yes
- [ ] Maybe, if (anonymization, just some variables, etc)?
- [ ] No, why?

H9. **Can the dataset and its metadata be shared with a specific partner of DECIDE consortium?**

- [ ] Yes
- [ ] Maybe, if (anonymization, just some variables, etc)?
- [ ] No, why?

H10. **Can the dataset and its metadata be shared with all members of the consortium under the DECIDE joint controller agreement?**

- [ ] Yes
- [ ] Maybe, if (anonymization, just some variables, ect) ?
- [ ] No, why?

H11. Can the dataset and its metadata be reused by another researcher outside of DECIDE consortium?

- Yes ☐
- Maybe, if (anonymization, just some variables, ect) ? ☐
- No, why? ☐

H12. In general, what would be your main barriers to data sharing?

Section I: Sharing dataset's full structure

I1. **To fully create opportunities without sharing the full data set, are you willing to send a full data set description in English (if you can) to us (DECIDE work package 1 leaders) by mail (camille.delavenne@ausvet.com.au)?**

This information would allow researchers to propose ways to aggregate the information to create anonymity, hence potentially creating further opportunities to share data in compliance with GDPR legislation.

If you answer yes to this question we will come back to you to ensure that you have no difficulties sharing the information.

This full description will depend on your dataset's structure. For example, if the dataset is structured similarly to a relational database, we would welcome a database diagram. Independently of the dataset's structure, a full description of the dataset should at least contain a variable dictionary and a description of every table contained in the dataset.

If you do not have a written document describing your dataset, you can use the formatted example (one sheet per table) in the following link: "open this link in a new tab" or copy it in your browse (*https://docs.google.com/spreadsheets/d/1aAW56SjkLS6lVP1bA3IPJbis18Xee-Kl/edit?usp=sharing&ouid=117166247232532251655&rtpof=true&sd=true)*

Yes ☐

No ☐

I2. **Are you also willing to share the dataset's metadata by email (camille.delavenne@ausvet.com.au)?**

If you answer yes to this question we will come back to you to ensure that you have no difficulties sharing the information.

When answering the previous questions, you potentially identified documents containing information about the dataset's governance, management, structure, and subject, those are also considered as metadata.

Yes ☐

No ☐

Section J: Sharing expectations

J1. What are your expectations when sharing this dataset for the DECIDE H2020 project?

Section K: Questionnaire's result

K1. Can the information in this questionnaire and all metadata information be available for other members of DECIDE consortium?

Yes ☐

Yes after anonymization ☐

No ☐

Other ☐

Other

K2. Can the information in this questionnaire be used in a publication after anonymization?

If yes, we will keep you informed and include you in the authorship discussion.

Yes ☐

No ☐

K3. Can the questionnaire results be accessible for researchers to re-use? (excluding personal data)

Yes ☐

No ☐

K4. **This questionnaire is ending, did you have any comments you wanted to leave us?**

We would like to thank you deeply for completing this questionnaire and we give you feedback on our results as soon as possible.
